# Aging in the dermis: Fibroblast senescence and its significance

**DOI:** 10.1111/acel.14054

**Published:** 2023-12-01

**Authors:** Jing Zhang, Haoyue Yu, Mao‐Qiang Man, Lizhi Hu

**Affiliations:** ^1^ Immunology Department, Key Laboratory of Immune Microenvironment and Disease (Ministry of Education) Tianjin Medical University Tianjin China; ^2^ Dermatology Hospital Southern Medical University Guangdong China; ^3^ Department of Dermatology University of California San Francisco and Veterans Affairs Medical Center San Francisco California USA

**Keywords:** dermis, ECM, fibroblast, inflammation, senescence

## Abstract

Skin aging is characterized by changes in its structural, cellular, and molecular components in both the epidermis and dermis. Dermal aging is distinguished by reduced dermal thickness, increased wrinkles, and a sagging appearance. Due to intrinsic or extrinsic factors, accumulation of excessive reactive oxygen species (ROS) triggers a series of aging events, including imbalanced extracellular matrix (ECM) homeostasis, accumulation of senescent fibroblasts, loss of cell identity, and chronic inflammation mediated by senescence‐associated secretory phenotype (SASP). These events are regulated by signaling pathways, such as nuclear factor erythroid 2‐related factor 2 (Nrf2), mechanistic target of rapamycin (mTOR), transforming growth factor beta (TGF‐β), and insulin‐like growth factor 1 (IGF‐1). Senescent fibroblasts can induce and accelerate age‐related dysfunction of other skin cells and may even cause systemic inflammation. In this review, we summarize the role of dermal fibroblasts in cutaneous aging and inflammation. Moreover, the underlying mechanisms by which dermal fibroblasts influence cutaneous aging and inflammation are also discussed.

## Graphical abstract

This mini review discusses aging, inflammation, cellular senescence, and their orchestration in cutaneous aging, recognizing fibroblast senescence as central player and inflammation response as key mediator in these processes. We delve into the cascade effects of stress, senescence, SASP, and related signaling in fibroblasts, elucidating how they contribute to the amplification of inflammation and ultimately lead to the appearance and functional alterations observed in aged skin.

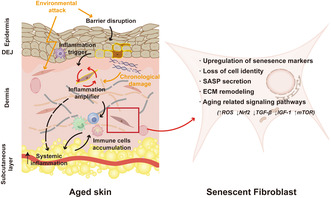

**Abbreviations**
C/EBPβ: CCAAT/enhancer‐binding protein beta
CDK: cyclin‐dependent kinases
CTGF/CCN2: connective tissue growth factor
ECM: extracellular matrix
EVs: extracellular vesicles
GATA4: GATA‐ binding protein 4
GM‐CSF: granulocyte‐macrophage colony‐stimulating factor
HO‐1: heme oxygenase 1
IFNγ: interferon gamma
IGF‐1: insulin‐like growth factor 1
JAK‐STAT: Janus kinase‐signal transducer and activator of transcription
KGF: keratinocytes growth factors
MAPK: mitogen‐activated protein kinase
MMPs: matrix metalloproteinases
mTOR: mechanistic target of rapamycin
NF‐κB: nuclear factor kappa‐light‐chain‐enhancer of activated B cells
NQO1: NAD(P)H quinone dehydrogenase 1
Nrf2: erythroid 2‐related factor 2
OIS: oncogene‐induced senescence
ROS: reactive oxygen species
SAASP: skin aging‐associated protein
SASP: senescence‐associated secretory phenotype
SA‐β‐gal: β‐galactosidase
SOD: superoxide dismutase
TGF‐β: transforming growth factor‐ beta
TIMP: tissue inhibitors of MMPs
TNFα: tumor necrosis factor‐ alpha
UV: ultraviolet

## 1 INTRODUCTION

Aging is a complex process resulting in decline in functions of tissues/organs. This event is driven by a variety of intrinsic and extrinsic factors, such as DNA damage accumulation (d’Adda di Fagagna, 2008), shortened telomeres (Olovnikov, 1996), mitochondrial dysfunction (Wiley et al., 2016), and exposure to other environmental stressors (Kammeyer & Luiten, 2015). These factors direct the cells to enter a state of irreversible growth arrest known as senescence (Colavitti & Finkel, 2005; Coppé et al., 2008). Although senescent cells are no longer able to divide, they remain metabolically active and can secrete a mixture of molecules known as senescence‐associated secretory phenotype (SASP) that contribute to inflammation via autocrine and paracrine mechanisms (Rodier et al., 2009).

**FIGURE 1:**
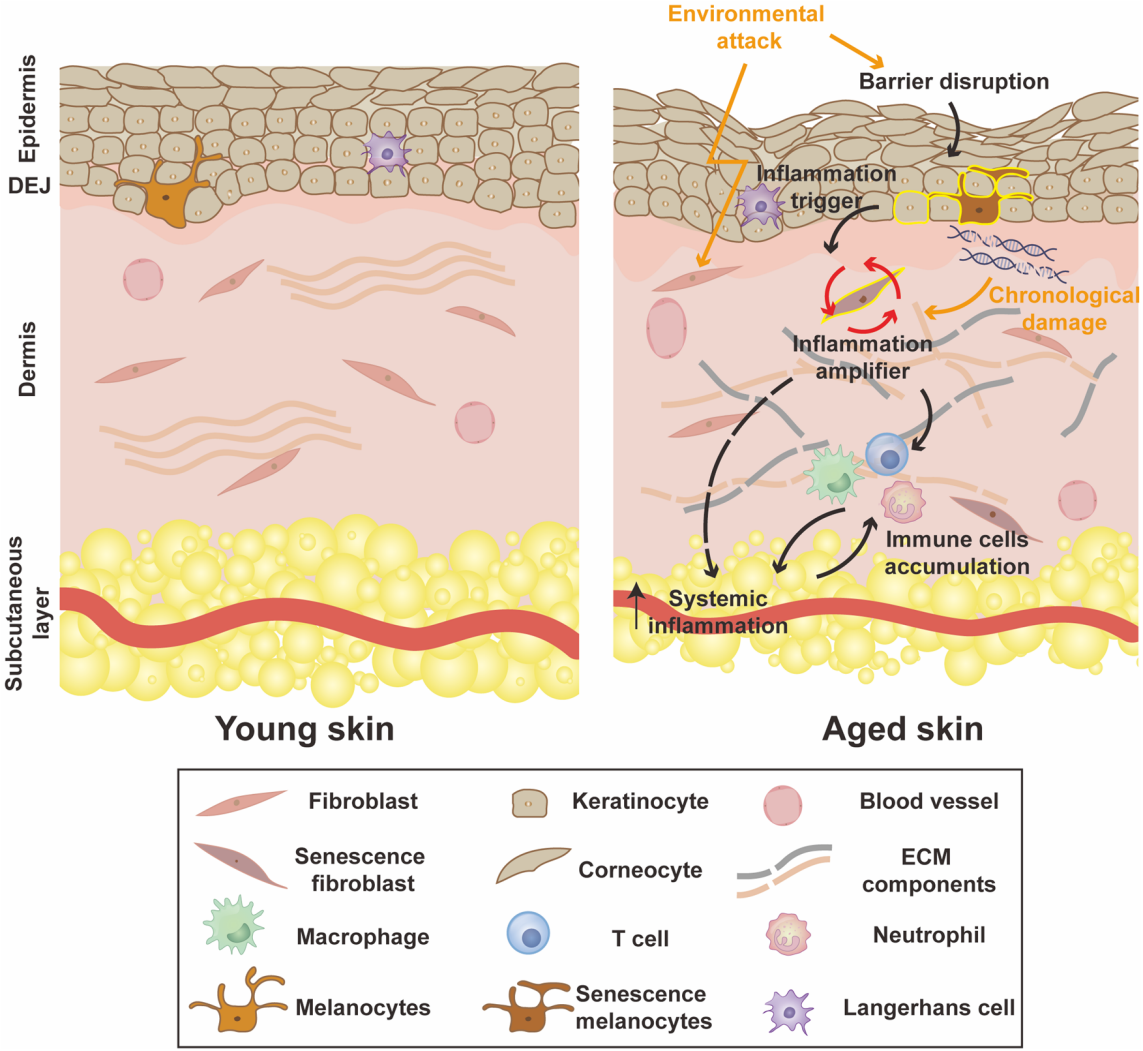
Schematic representation of dermal fibroblasts as a crucial amplifier in skin aging and inflammation. Aged skin is characterized by a dysfunctional epidermal barrier, thinner dermis, accumulated senescent fibroblasts, excessive immune cells, and fragmented ECM components. Chronological changes in cutaneous functions and external stressors induce skin cells such as keratinocytes and melanocytes to initiate skin senescence. Fibroblasts can receive aging signals through paracrine resources, such as neighboring cells like keratinocytes and melanocytes, or through autocrine secretion of SASP induced by intrinsic or extrinsic factors. Senescent fibroblasts play a key role in exacerbating cutaneous aging and amplifying inflammation by producing cytokines, chemokines, and other factors. This attracts and activates immune cells, leading to inflammation not only in the skin but also potentially in other parts of the body.

Skin, primarily comprising the epidermis and the dermis, serves as a physical barrier protecting the body from external insults. The epidermis, a stratified squamous epithelium, is mainly composed of keratinocytes, melanocytes, and Langerhans cells (Liu et al., 2021; Tang et al., 2020). Situated beneath the epidermis, the dermis plays a crucial role in structure and function. It contains an abundant extracellular matrix (ECM) produced by fibroblasts and houses various cell types due to its diverse structures, including vasculature, nerves, sweat glands (Hosseini et al., 2022; Weng et al., 2020) (Figure 1). Constantly exposed to external insults, the skin undergoes significant changes through our lifetime that differentiate the skin of a child from that of an older adult. These changes are caused by a combination of intrinsic aging, also known as chronological aging, and extrinsic aging induced by environmental factors, including air pollution, poor nutrition, smoking, and ultraviolet (UV) light. Both epidermal keratinocyte and dermal fibroblast senescence contribute to the skin aging (Fitsiou et al., 2021; Gruber et al., 2020; Wang et al., 2020). The role of keratinocytes in aging and inflammation has been well summarized by others (Wang et al., 2020). In this review, we focus on the dermal fibroblasts in skin aging and propose the crucial role of dermal fibroblasts in inflammation.

## 2 MECHANISMS OF DERMAL AGING

### 2.1 Excessive ROS, a driving force of dermal aging

Skin aging involves the intricate interplay of various mechanisms and multiple causal processes, such as nuclear DNA damage (García‐Beccaria et al., 2014; Rodier et al., 2009), generation of excessive reactive oxygen species (ROS), and mitochondria dysfunction (Kaneko et al., 2012; Krutmann & Schroeder, 2009; Yang et al., 1995). Among these mechanisms, the oxidative stress theory holds a prominent position, which emphasizes ROS as driving force of aging. ROS, also known as free radicals or oxidants, possess diverse properties and biological functions, ranging from oxidative metabolism to cell signaling (Ray et al., 2012; Sies & Jones, 2020). Under normal physiological conditions, ROS are generated as natural byproducts of cell metabolism. However, as we age, ROS assume a dual role, acting as both primary triggers and critical consequences of skin aging. Intrinsic factors such as mitochondrial dysfunction (Sreedhar et al., 2020), along with external factors like UV radiation (Chaiprasongsuk & Panich, 2022), and other stressors, synergistically augment ROS production and retard ROS removal by antioxidants. Overtime, the accumulation of ROS proceeds to oxidize lipids, nucleic acids, proteins, and organelles, leading to the cell and tissue dysfunction (Lee & Wei, 2001; Rinnerthaler et al., 2015). It is worth noting that a vicious circle exists between oxidative stress and inflammation during aging. ROS serve as signaling molecules that trigger inflammatory responses, and inflammatory cytokines and chemokines in turn generate more ROS and free radicals (Kammeyer & Luiten, 2015; Zinovkin et al., 2022).

The pathogenic role of accumulated ROS in chronological aging (Papaccio et al., 2022; Poljšak et al., 2012; Tu & Quan, 2016) is supported by a significant increase in ROS levels in aged human fibroblasts in vitro (Kozieł et al., 2011) and in aged rat skin in vivo (Tahara et al., 2001). In addition, a line of evidence also supports the role of excessive ROS in skin photoaging in vitro and in vivo (Jurkiewicz & Buettner, 1996; Li et al., 2018; Masaki et al., 1995; Yasui & Sakurai, 2000). In addition, inhibition of ROS production in these cells reduces the number of cells entering cell‐cycle arrest (Cavinato et al., 2017). The driving force of ROS in skin aging and fibroblast senescence is evident in a mouse model with fibroblast specific superoxide dismutase‐2 (SOD2) deficiency, where mitochondrial superoxide anions accumulate in fibroblasts. Selective SOD2 deficiency results in a severe and accelerated skin aging phenotype, characterized by reduced collagen and dermal thickness, diminished resilience, enhanced DNA damage, and accumulation of senescent fibroblasts (Weyemi et al., 2012). Excessive ROS in fibroblasts can activate several signaling pathways, including mitogen‐activated protein kinase (MAPK), nuclear factor kappa‐light‐chain‐enhancer of activated B cells (NF‐κB), TGF‐β, and mTOR, leading to the accumulation of senescent fibroblasts, induction of chronic inflammation, and disruption of ECM homeostasis (Ansary et al., 2021; Bang et al., 2021; Chen et al., 2022; Gu et al., 2020).

### 2.2 Role of senescent dermal fibroblasts in skin aging

**FIGURE 2:**
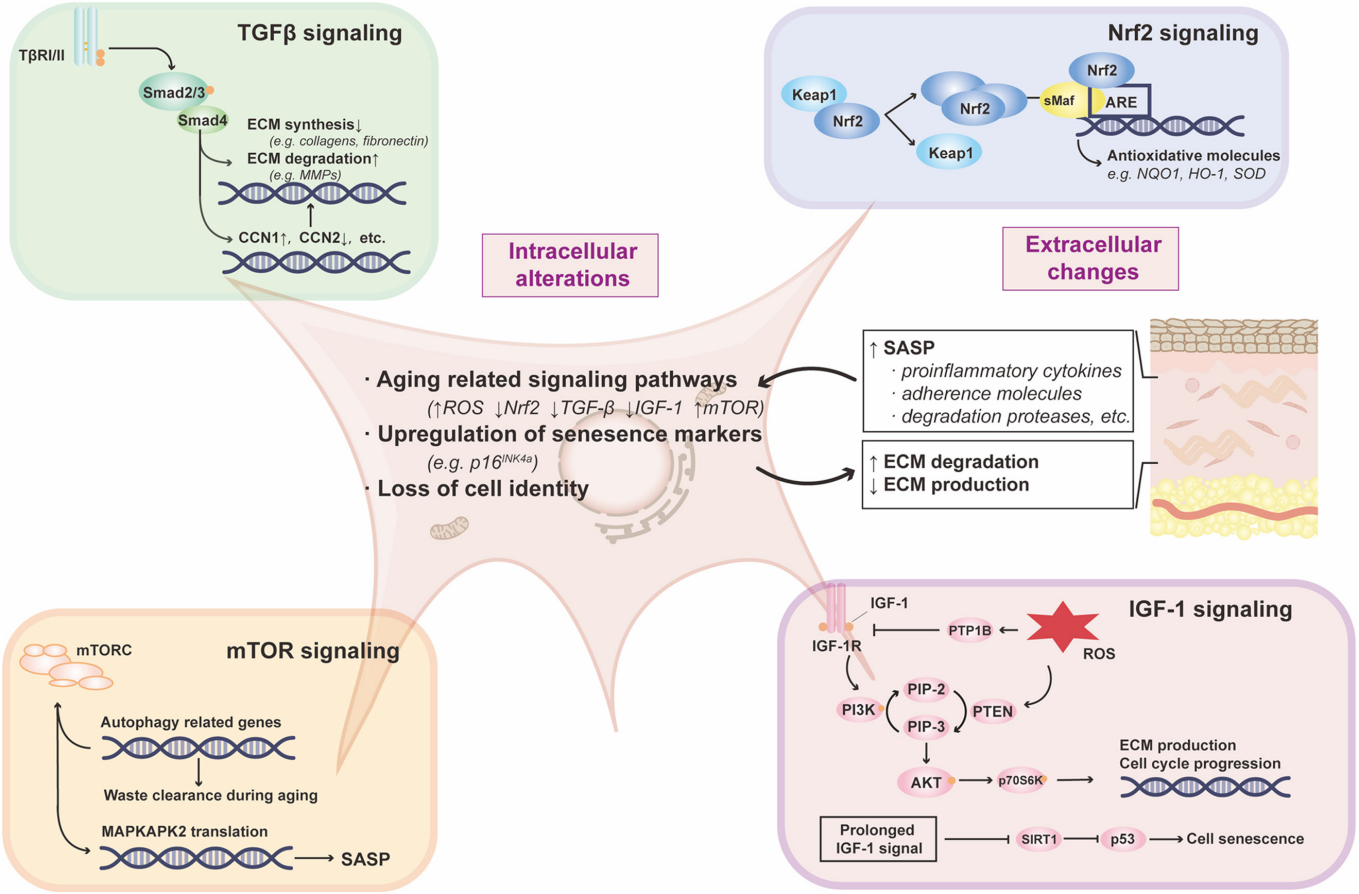
Extracellular and intracellular alterations occur during dermal aging. Intrinsic and extrinsic factors induce alterations in age‐related pathways in dermal fibroblasts, including Nrf2 signaling, TGF‐β signaling, IGF‐1 signaling, and mTOR signaling. Additionally, an upregulation of senescence markers and loss of cell identity are also evident during fibroblast aging. The senescent fibroblasts secrete multiple SASP factors that contribute to inflammatory response and ECM dysregulation.

Senescence inducers, including stressors (ROS, DNA damage, irradiation), telomere attrition, and mitochondria dysfunction, increase the activity of cyclin‐dependent kinases (CDK) inhibitor proteins, resulting in cell‐cycle arrest. In comparison with young skin (18–29 year), the total number of fibroblasts is reduced by approximately 35% in aged skin (>80 year) (Varani et al., 2000), while the number of senescent fibroblasts is increased with age, as evidenced by a significant increase in p16^INK4a^ positive cells (a senescent cell marker that encodes an inhibitor of CDK4/6) in the dermis of the aged human skin (Ogata et al., 2021; Ressler et al., 2006; Waaijer et al., 2012). The number of p16^INK4a^ positive cells is also correlated with wrinkle formation and elastic morphological changes (Waaijer et al., 2016). Other classical senescence biomarkers, such as p21^CIP1^, p53, and β‐galactosidase (SA‐β‐gal), are upregulated, and lamin B1 is downregulated in aged or UV‐irradiated fibroblasts (Chen et al., 2008; Dimri et al., 1995; McCart et al., 2017; Ravelojaona et al., 2009; Wang et al., 2017). Like other senescent cells, senescent fibroblasts also experience proliferation arrest, yet remain viable due to their low propensity for apoptosis and inefficient removal by the immune system, causing them to persist in the stroma. An excessive buildup of senescence fibroblasts contributes significantly to skin aging, as these fibroblasts display loss of cell identity (Solé‐Boldo et al., 2020; Salzer et al., 2018; Zou et al., 2021), enhanced release of SASP (Rodier et al., 2009; Waldera‐Lupa et al., 2014), and dysfunction of ECM homeostasis (Treiber et al., 2011; Wlaschek et al., 2021) (Figure 2). Consequently, senescence spreads from cells to cells, fueling the process of dermal aging (da Silva et al., 2019).

### 2.3 Dermal SASP in skin aging

SASP refers to a mixture of molecules (including cytokines, matrix metalloproteinases(MMPs), miRNAs, chemokines, growth factors, and small‐molecule metabolites) released by senescent cells, which have immunoregulatory effects and impact the proliferation and motility of non‐senescent cells. Proteins that are involved in matrix degradation (MMP1, MMP3, MMP10, MMP14, etc.) and proinflammatory processes, such as interleukin‐1β (IL‐1β), IL‐8, IL‐15, interferon gamma (IFNγ) have been found in both skin aging‐associated protein (SAASP) and canonical SASP, suggesting shared senescent traits across different tissue contexts (Waldera Lupa et al., 2015). Moreover, unique expression patterns of proteins related to metabolism and adherence junction interactions were found in SAASP (Waldera Lupa et al., 2015). Epilipomics have revealed the presence of SASP lipids in aged dermal fibroblasts, with lysophosphatidylcholines as a pleiotropic factor that can elicit chemokine release in non‐senescent fibroblasts and interfere with macrophage activity (Narzt et al., 2021).

### 2.4 Inflammatory factors in SASP bridges inflammatory communication between cells

The inflammatory factors in SASP play a dual role in cellular senescence. In an autocrine manner, they reinforce the senescence and inflammatory state of fibroblast themselves (Acosta et al., 2008; Kumar et al., 1992; Wlaschek et al., 1994). Simultaneously, they can act in a paracrine manner to induce inflammatory response in surrounding cells (Acosta et al., 2013; Ghosh & Capell, 2016; Wlaschek et al., 2021). Senescent human dermal fibroblasts produce extracellular vesicles (EVs) which are less supportive for keratinocyte differentiation and barrier function, but contain higher levels of IL‐6 compared to EVs from young dermal fibroblasts (Choi et al., 2020). SASP cytokines and chemokines, such as IL‐1, IL‐8, and tumor necrosis factor alpha (TNFα), attract immune cells like macrophages, neutrophils, and T cells. SASP secreted by senescent fibroblasts can hinder macrophage‐dependent clearance and potentiate further accumulation of senescent cells (Ogata et al., 2021). Thus, SASP can act as a bridge between fibroblasts and other skin cells during aging. The classical transcription factors controlling SASP secretion include p53, NF‐κB, CCAAT/enhancer‐binding protein beta (C/EBPβ), Janus kinase‐signal transducer and activator of transcription (JAK–STAT), and GATA binding protein 4 (GATA4) (Huggins et al., 2013; Kang et al., 2015; Salminen et al., 2012; Xu et al., 2015). Dermal fibroblasts modulate SASP secretion and senescence phenotypes via signaling pathways, such as Nrf2, mTOR, TGF‐β, and IGF‐1 (Figure 2), which will be discussed later.

### 2.5 ECM‐modifying enzymes in SASP impair ECM homeostasis

Aged dermis exhibits prominent clinical features, including decreased dermis thickness, reduced resilience, and mechanical force with wrinkled and flabby appearance (Farage et al., 2008). These changes are related to the loss of ECM components during aging, due to a reduced synthesis and enhanced degradation of ECM in aged fibroblasts (Autio et al., 1994; Varani et al., 2006). SASP components like MMPs, which can directly cleavage collagen fibrils, play a significant role in ECM degradation during aging. Previous studies have shown that the levels of MMPs, including MMP1, MMP2, and MMP9, are increased in the aged dermis and cultured fibroblasts derived from aged participants (Qin et al., 2017; Quan et al., 2013). Overexpression of hMMP1 induces aging phenotypes in ex vivo 3D human skin organ culture (Xia et al., 2013) and in vivo mice model (Quan et al., 2023). Additionally, the elevated levels of MMPs are paralleled by a reduction of tissue inhibitors of MMPs (TIMPs) in the aged skin, leading to an imbalance of MMPs/TIMPs and progressive collagen fragmentation (Yokose et al., 2012). TIMP‐1 overexpression protects ECM against degradation and elasticity reduction induced by chronic UVB exposure, while the TIMP‐1 neutralizing antibody acts in an opposite way (Yokose et al., 2012). Besides, fibroblasts of the aged skin decrease the production of ECM, especially the major collagen network components, such as collagen type I and III (Brinckmann et al., 1995; Lovell et al., 1987). This process is primarily a result of the reduction in TGF‐β signaling, which will be discussed in detail later.

## 3 KEY SIGNALING PATHWAYS INVOLVED IN DERMAL FIBROBLAST SENESCENCE

Several signaling pathways are involved in the dermal aging. First, oxidative stress has long been recognized as a key regulator of aging process. Activation of Nrf2 increases the expression of antioxidation‐related factors, such as heme oxygenase 1 (HO‐1), NAD(P)H quinone dehydrogenase 1 (NQO1), and superoxide dismutase (SOD), to protect against oxidative stress burden and inflammation (Figure 2). Nrf2 activity is reduced during photoaging and chronological aging of human fibroblasts (Kapeta et al., 2010) and murine fibroblasts (Jódar et al., 2011), while silencing of Nrf2 induces premature aging (Kapeta et al., 2010). Accordingly, enhancement of Nrf2 signaling can attenuate aging and inflammation in dermal fibroblasts (Guo et al., 2022; Hseu et al., 2019; Lee et al., 2022; Sklirou et al., 2017). Thus, downregulation of Nrf2 signaling pathway can contribute to dermal aging.

Second, multiple studies indicate the pivotal role of TGF‐β in dermal aging (Figure 2). Physiologically, aged dermis exhibits lower content of ECM (de Bengy et al., 2022; Fisher et al., 1996). TGF‐β signaling can enhance ECM gene expression (collagens, fibronectin, decorin, versican), while inhibiting ECM degradation by downregulation of MMPs and upregulation of TIMPs (Quan & Fisher, 2015; Verrecchia et al., 2001). Either oxidative stress (He et al., 2014) or UV irradiation (Quan et al., 2004) impairs TGF‐β signaling pathway in fibroblasts, resulting in decreased expression of downstream targets, including connective tissue growth factor (CTGF/CCN2) and type I collagen (He et al., 2014; Quan et al., 2004, 2010). Knockdown of TβRII (Quan et al., 2004) or Smad3 (Purohit et al., 2016) impairs collagen synthesis while overexpression of TβRII rescues UV‐induced loss of collagen via activation of TGF‐β signaling. Impaired TGF‐β signaling alters expression levels of CCN1 in dermal fibroblasts of mice, resembling aged skin manifested by wrinkled appearance and disruption of collagen network (Quan et al., 2021). In addition, reduced fibroblast size, a characteristic of dermal fibroblasts in the aged skin, is associated with reduced expression levels of TβRII and diminished ECM production in the aged human skin (Fisher et al., 2016). Hence, abrogated TGF‐β signaling pathway is associated dermal aging.

Moreover, evidence also suggests the involvement of IGF‐1 signaling pathway in dermal aging (Figure 2). IGF‐1 signaling begins with the phosphorylation of IGF‐1Rβ, followed by a series of activation of downstream pathways such as PI3K/AKT/p70S6K or Ras/Raf/MEK/ERK, to regulate cell cycle and protein biosynthesis (Hakuno & Takahashi, 2018; Salminen & Kaarniranta, 2010). IGF‐1 pathway can be deregulated by age‐associated superoxide anion accumulation, leading to limited fibroblast proliferation and collagen deposition (Singh et al., 2015). Transcription factor JunB‐induced inhibition of IGF‐1 promotor activation decreases the levels of IGF‐1 and its downstream PI3K/AKT pathway effectors, resulting in disruption of the metabolic and structural niches of skin stem cells, consequently leading to exacerbation of skin aging (Maity et al., 2021). Aging‐related reduction in circulating IGF‐1 levels and impaired IGF‐1 signaling likely contribute to the atrophy of skin, muscle, and bone in the elderly (Gallagher & LeRoith, 2011). However, long‐term treatment of primary human skin fibroblasts with IGF‐1 induces a premature senescence phenotype (Nagaraj et al., 2022; Tran et al., 2014). Thus, further studies are needed to elucidate the role of IGF‐1 signaling in dermal aging.

Additionally, the contribution of mTOR signaling pathway to dermal aging has been well appreciated because of its regulatory role in cellular metabolism and autophagy (Figure 2). Previous study demonstrated that activation of autophagy promotes the degradation of oxidized metabolites and inhibition of photoaging via inhibition of PI3K/AKT/mTORC1 signaling (Chen et al., 2022; Wang et al., 2019). In contrast, loss of autophagy or upregulation of mTOR signaling contributes to photoaging (Chen et al., 2022; Lim et al., 2020; Wang et al., 2019). Moreover, mTOR can also suppress SASP production via MAPKAPK2 translation in oncogene‐induced senescence (OIS) (Herranz et al., 2015). Treatment with pan‐mTOR inhibitor, AZD8055, can modify the senescence phenotypes in skin fibroblasts (Walters et al., 2016). Similarly, the mTOR inhibitor Rapamycin can decrease the expression p16^INK4a^ and ameliorate SASP secretion in senescent fibroblasts, and lead to visible improvement in aging skin appearance (Chung et al., 2019; Herranz et al., 2015; Laberge et al., 2015). Therefore, dermal aging is linked to activation of mTOR signaling pathway. Inhibition of mTOR signaling pathway can exhibit antiaging benefit.

## 4 DERMAL FIBROBLASTS AS A POTENTIAL AMPLIFIER TO SKIN AGING AND INFLAMMATION

Chronological changes in cutaneous functions and external stressors induce telomere attrition, ROS accumulation, DNA damage, and mitochondrial dysfunction in dermal fibroblasts, resulting in diverse forms of senescence (Franco et al., 2022). Senescent fibroblasts exhibit irreversible cell‐cycle arrest and release SASP, which distinguishes them from other non‐proliferative cells. SASP, comprised of various components, plays multiple roles in aging. It builds up chronic inflammation through cytokines and chemokines, impairs proliferation by disrupting the release of growth factors, and remodels the ECM through enhanced activation of proteolytic enzymes (Wlaschek et al., 2021). SASP is not only a byproduct of senescent fibroblasts, but it also serves as a messenger to reinforce senescence in both paracrine and autocrine manners (Ghosh & Capell, 2016; Tasdemir & Lowe, 2013; Wlaschek et al., 2021). The cascading effects of stress, senescence, and SASP signaling in fibroblasts might contribute to the development of aging‐associated cutaneous abnormalities, including wrinkles, loss of volume, and elasticity of collagen (Imokawa, 2009; Shuster et al., 1975) impaired wound healing (Mahmoudi et al., 2019; Thanapaul et al., 2022), and might related to the increased risk of inflammatory dermatoses in the elderly (Wang et al., 2020).

The age‐related cutaneous dysfunction could serve as an initiator of inflammation, while the development and sustained inflammation during skin aging is resultant from a coordinated effort among various skin cells, including keratinocytes, fibroblasts, melanocytes, and innate/adaptive immune cells (Figure 1). Recent research has explored the communication between keratinocytes and fibroblasts and has proposed the existence of a positive feedforward loop. Specifically, keratinocyte‐produced IL‐1 appears to play a crucial role in inducing fibroblasts to produce cytokines (IL‐1, IL‐6, IL‐8) and growth factors (keratinocytes growth factors (KGF), granulocyte‐macrophage colony‐stimulating factor (GM‐CSF)). In turn, these factors secreted by fibroblasts regulate the biological functions of keratinocytes, including proliferation, differentiation, and cytokine production (Russo et al., 2020). Additionally, melanocytes are crucial resources of aging signals. Chronological aging and chronic UVR exposure promotes the accumulation of senescent melanocytes, which exhibit disrupted glycolytic metabolism and telomere dysfunction (Park et al., 2023; Victorelli et al., 2019). The accumulated senescent melanocytes produce SASP, which induces paracrine telomere dysfunction transmission in neighboring cells like fibroblasts and keratinocytes through the IP‐10‐CXCR3‐ROS signaling (Victorelli et al., 2019). Thus, while keratinocytes and melanocytes may act as triggers of inflammation, fibroblasts may serve as the amplifiers of inflammation (Figure 1). The SASPs generated by initiators and amplifiers further activate resident immune cells and recruit circulating immune cells to deteriorate inflammation (Fitsiou et al., 2021).

Recent evidence from scRNAseq of chronological aging and photoaging in humans and mice highlight fibroblasts as a major responder in age‐related inflammation. For instance, Lin et al. demonstrated that UV‐irradiated mouse skin mainly induces inflammatory responses in fibroblasts (Lin et al., 2022). The transcriptomic atlas suggests that fibroblasts exhibit the highest level of aging‐related transcriptional variability among all the identified skin cell types (Zou et al., 2021). These observations align with the defective ECM and thinned dermis observed in aged human skin. Therefore, it is important to focus on regulating the amplifier of inflammation in fibroblasts to control inflammation, restore ECM homeostasis, and maintain a youthful and healthy appearance. However, further research is required to validate the link between dermal fibroblast function and inflammation.

## 5 CONCLUDING REMARKS

As we are aging, everyone will eventually face a problem of skin aging. Fibroblast senescence is attributed to alterations in multiple signaling pathways, including Nrf2, TGF‐β, IGF‐1, and mTOR. Dermal fibroblast senescence can induce and exacerbate cutaneous inflammation, while sustained cutaneous inflammation can lead to chronic systemic inflammation, that is, inflammaging (Franco et al., 2022; Pilkington et al., 2021). Thus, dermal fibroblast senescence can potentially and negatively impact overall health of human being. Therefore, attenuation of skin aging, including fibroblast senescence, can benefit, at least, some health conditions in the elderly. Because multiple mechanisms can contribute to fibroblast senescence, development of ideal approaches to prevent/treat dermal aging is still a challenge although applications of antioxidants show some benefits (Boo, 2022; Lee et al., 2022; Lephart, 2016). Additionally, further studies are needed to delineate the link between fibroblast senescence and inflammaging.

## AUTHOR CONTRIBUTIONS

Jing Zhang: Conception and design, manuscript writing, figure design. Haoyue Yu: Conception and design, manuscript writing, figure design. Mao‐Qiang Man: Manuscript revision. Lizhi Hu: Conception and design, financial support, manuscript revision, final approval of manuscript.

## FUNDING INFORMATION

This research was funded by National Natural Science Foundation of China, grant number NSFC 81972962 and 82273563 to L.H.

## CONFLICT OF INTEREST STATEMENT

All authors declared no potential conflict of interest.

## Data Availability

Data sharing is not applicable to this article as no new data were created or analyzed in this study.
